# CircCSNK1G3 up‐regulates miR‐181b to promote growth and metastasis via TIMP3‐mediated epithelial to mesenchymal transitions in renal cell carcinoma

**DOI:** 10.1111/jcmm.15911

**Published:** 2021-02-09

**Authors:** Wen Li, Yang‐Yi‐Yan Song, Ting Rao, Wei‐Min Yu, Yuan Ruan, Jin‐Zhuo Ning, Xiao‐Bing Yao, Song‐Yi‐Sha Yang, Fan Cheng

**Affiliations:** ^1^ Department of Emergency Renmin Hospital of Wuhan University Wuhan China; ^2^ Department of Pharmacy Renmin Hospital of Wuhan University Wuhan China; ^3^ Department of Urology Renmin Hospital of Wuhan University Wuhan China; ^4^ College of Pharmacy Hubei University of Chinese Medicine Wuhan China

**Keywords:** CircCSNK1G3, epithelial to mesenchymal transition (EMT), miR‐181b, renal cell carcinoma, TIMP3

## Abstract

Renal cell carcinoma (RCC) is the most common form of kidney cancer, with a high recurrence rate and metastasis capacity. Circular RNAs (circRNAs) have been suggested to act as the critical regulator in several diseases. This study is designed to investigate the role of circCSNK1G3 on RCC progression. We observed a highly expression of circCSNK1G3 in RCC tissues compared with normal tissues. The aberrantly circCSNK1G3 promoted the tumour growth and metastasis in RCC. In the subsequent mechanism investigation, we discovered that the tumour‐promoting effects of circCSNK1G3 were, at least partly, achieved by up‐regulating miR‐181b. Increased miR‐181b inhibits several tumour suppressor gene, including CYLD, LATS2, NDRG2 and TIMP3. Furthermore, the decreased TIMP3 leads to the enhanced epithelial to mesenchymal transition (EMT) process, thus promoting the cancer metastasis. In conclusion, we identified the oncogenic role of circCSNK1G3 in RCC progression and demonstrated the regulatory role of circCSNK1G3 induced miR‐181b expression, which leads to TIMP3‐mediated EMT process, thus resulting in tumour growth and metastasis in RCC. This study reveals the promise of circCSNK1G3 to be developed as a potential diagnostic and prognostic biomarker in the clinic. And the roles of circCSNK1G3 in cancer research deserve further investigation.

## 1 INTRODUCTION

Kidney cancer accounts for at least 2% of malignant diseases, and its incidence has a rising rate of about 2%‐3% per decade worldwide.^1^, ^2^, ^3^ Renal cell carcinoma (RCC) is the most common form of kidney cancer in adults. In the clinic, approximately 20% of the patients are diagnosed with advanced RCC and nearly 30% of local RCC patients relapsed and underwent metastasis after surgery.^4^ Although chemotherapy and other therapies have developed rapidly in recent years, the current clinically preferred treatment is still surgery.^5^ In addition, renal cell carcinoma is a genetically and histopathologically heterogeneous disorder and about 3% of cases of RCC are recognized as having a genetic basis.^6^ Pathological researches indicated the complexity of the molecular mechanism of RCC in terms of genomics.^7^, ^8^ Therefore, further comprehension of the underlying molecular mechanism about renal cell carcinoma progression is urgently needed. It is of paramount importance to identify the prognostic biomarkers and therapeutic targets for RCC.

A poorly characterized component of RCC transcriptome is circular transcripts (circRNAs). Circular RNA is originally thought to be the noise of the genomic. But recently, circular RNAs have been reported to have crucial biological efficacies.^9^ Multiple circular RNAs have been shown to be highly expressed in a tissue‐specific or cell type‐specific manner.^10^, ^11^ More importantly, many circular RNAs are involved in human epithelial‐mesenchymal transition (EMT) process.^12^ These findings suggested the functionality of circRNAs. In fact, studies have demonstrated the regulatory potency and tumour‐promoting properties of circular RNAs in multiple human diseases.^13^, ^14^ Accumulated evidence showed circRNAs participated in a variety of carcinoma activities, such as proliferation, apoptosis and the like.^15^ Altered circRNAs expression leads to abnormally gene expression which contributes to the cancer progression.^16^, ^17^ However, the overall pathophysiological contribution of circular RNAs in renal cell carcinoma is still largely unknown. In this research, we characterized one circular RNA circCSNK1G3 which was first identified to act as an oncogenic role and promote cell growth by interacting with miR‐181 in prostate cancer.^18^ The studies on circCSNK1G3 are lacking so far, and the efficacy of circCSNK1G3 in renal cell carcinoma is also unclear yet. In this study, the functions and mechanisms of circCSNK1G3 in RCC progression were investigated.

In addition, circRNAs often function by interacting with microRNAs (miRNAs),^19^ a type of non‐coding RNAs with 20‐22 nucleotide that regulates gene expression post‐transcriptionally through controlling mRNA translation efficiency.^20^, ^21^ MiRNAs were distributed in several cancers and were widely studied because of the therapeutic potential in the clinic.^22^ Recently, many researches demonstrated that circular RNAs regulated cancer progression via micro RNAs. For example, circRBM33 promotes cell proliferation, migration and invasion in gastric cancer cells by targeting miR‐149,^23^ and circular RNA circFNDC3B prevents renal carcinoma via targeting miR‐99a.^24^ In this research, we found a positively regulatory effect of circCSNK1G3 on miRNA miR‐181b, a miRNA which was previously suggested to be abnormally expressed in a variety of human cancers such as breast cancer and non‐small cell lung cancer.^25^, ^26^ Previous studies have clarified the role of some miRNAs, as the tumour suppressor ^27^ or the tumour promoter,^28^ in renal cell carcinoma, but the efficacy of miR‐181b in renal cell carcinoma remains unknown. In this research, the role of miR‐181b in RCC was preliminarily confirmed. More important, the interaction between circCSNK1G3 and miR‐181b was identified, and the underlying mechanism by which the circCSNK1G3/miR‐181b axis regulated the development of renal cell carcinoma was clarified.

Overall, this research explored the role of circular RNA circCSNK1G3 in human renal cell carcinoma, the interaction between circCSNK1G3 and miR‐181b, as well as the underlying regulatory mechanism of circCSNK1G3 on the development of renal cell carcinoma. Further comprehension of the role of circCSNK1G3 and miR‐181b in renal cell carcinoma may be of great importance for early diagnosis and clinical targeted treatment of renal cell carcinoma.

## 2 METHOD

### 2.1 Cell culture and transfection

Human renal epithelial cell line 4120, renal tubular epithelial cell line HK‐2, embryonic kidney cell line 293FT and Human renal carcinoma cells 786‐O, Caki‐1, A498 and ACHN were purchased from the Type Culture Collection of the Chinese Academy of Sciences (Shanghai, China) and cultured in DMEM (Thermo Fisher Scientific.) with 10% FBS (Gibco) and 1% penicillin/streptomycin (Santa Cruz) at 37°C with 5% CO_2_.

For cell transfection, ACHN cells were transfected with plasmid containing miR‐181b inhibitor or miR‐181b mimics for miR‐181b knockdown or overexpression; shcircCSNK1G3 or pcDNA3.1‐circCSNK1G3 for circCSNK1G3 knockdown or overexpression using Lipofectamine 2000 reagent (Invitrogen). The shRNA oligo sequence was listed in Table 1. Plasmids used in this research were synthesized by GeneChem (Shanghai, China).

**TABLE 1:** ShRNA oligos for circRNAs

circCSNK1G3‐sh1	CCGGATCTGGAGCTCTCTATCAATACTCGAGTATTGATAGAGAGCTCCAGAT TTTTTG
circCSNK1G3‐sh2	CCGGTCTGGAGCTCTCTATCAATATCTCGAGATATTGATAGAGAGCTCCAGA TTTTTG

### 2.2 qRT‐PCR

Trizol (Invitrogen) was used to extract total RNA from cells. SuperScript III first‐strand synthesis system (Invitrogen) was used for reverse transcription. The qPCR was performed by SYBR‐Green PCR Master Mix (Thermo Fisher Scientific). Primers used in qPCR were manifested in Table 2.

**TABLE 2:** Primers used in qPCR

Primer Name	Primers	Sequences (5′‐3′)
CircCSNK1G3	F	GCACCACAGCTACATTTGGA
	R	GGAGCATGTTCATCCCATTC
mCSNK1G3	F	TGAGAGGCAGTCTTCCTTGG
	R	ACATAACGAAGATATGTTGCCATT
U6	F	CTCGCTTCGGCAGCACA
	R	AACGCTTCACGAATTTGCGT
miR‐181a	F	ACACTCCAGCTGGGAACATTCAACGCTGTCG
	R	GGTGTCGTGGAGTCGGCAATTCAGTTGAG
miR‐181b	F	CGACGAACATTCATTGCTG
	R	CAGTGCAGGGTCCGAGGTAT
miR‐181c	F	TTCTTCAACATTCAACCTGTCG
	R	TATCGTTGTACTCCAGACCAAGAC
CYLD	F	TCAGGCTTATGGAGCCAAGAA
	R	ACTTCCCTTCGGTACTTTAAGGA
LATS2	F	ACCCCAAAGTTCGGACCTTAT
	R	CATTTGCCGGTTCACTTCTGC
NDRG2	F	ACTTTGTGCGGGTTCATGTG
	R	CAGGACGCAAGGGATCATGTC
TIMP3	F	ACGCTGGTCTACACCATCAAGC
	R	CCGAAATTGGAGAGCATGTCG
E‐cadherin	F	TGCCCAGAAAATGAAAAAGG
	R	GTGTATGTGGCAATGCGTTC
N‐cadherin	F	GACAATGCCCCTCAAGTGTT
	R	CCATTAAGCCGAGTGATGGT
Vimentin	F	GAGAACTTTGCCGTTGAAGC
	R	TCCAGCAGCTTCCTGTAGGT
Snail	F	TTTACCTTCCAGCAGCCCTA
	R	CCTCATCTGACAGGGAGGTC
Slug	F	CTTTTTCTTGCCCTCACTGC
	R	ACAGCAGCCAGATTCCTCAT
Twist	F	GTCCGCAGTCTTACGAGGAG
	R	CCAGCTTGAGGGTCTGAATC
ZEB1	F	CAGCTTGATACCTGTGAATGGG
	R	TATCTGTGGTCGTGTGGGACT
GAPDH	F	CATGAGAAGTATGACAACAGCCT
	R	AGTCCTTCCACGATACCAAAGT

### 2.3 Fluorescence in situ hybridization (FISH)

FISH assay was performed as previously described^29^, ^30^ with minor modification. Biotin‐labelled probes specific to circCSNK1G3 were used in the hybridization.

### 2.4 Biotin‐coupled RNA pull‐down assay

The biotin‐coupled RNA pull‐down assay was performed as previously described.^29^ Briefly, the 3′‐end biotinylated miR‐181b mimics or circCSNK1G3 (RiboBio) were transfected into ACHN cells. The biotin‐coupled RNA complex was pulled down by incubating the cell lysates with streptavidin‐coated magnetic beads (Life Technologies). The abundance of circCSNK1G3 or miR‐181b in bound fractions was evaluated by qRT‐PCR analysis.

### 2.5 Luciferase report assay

The wild‐type (WT) and mutant genomic region of circCSNK1G3 sequence were inserted into pGL3 basic vector (Primega, Madison, WI). Plasmid containing WT or mutant circCSNK1G3 was cotransfected with miR‐181b mimics or corresponding control into ACHN cells using Lipofectamine 2000 reagent (Invitrogen).

### 2.6 MTT

Cells were transferred to a 96‐well plate and cultured for 1 day. 0.5% MTT (Solarbio, China) was added to the medium, and then, cells were cultured for 4 hours. The supernatant was discarded, and DMSO was added and shaking for 10 minutes on a shaker.

### 2.7 Colony formation assay

Cells were digested with trypsin and resuspended with culture medium following transfer into a 6‐well plate with about 300 cells per well. Then, cells were cultured at 37°C with 5% CO_2_ for 2‐3 weeks. Then, the cells were fixed with formaldehyde after washing with PBS and stained with crystal violet for observation.

### 2.8 Wound healing assay

Cells were transferred into 6‐well e‐plate and cultured for 1 day. A straight linear was made by pipette tip, and the cells were subsequently washed by 1 × PBS and cultured for 24 or 48 hours followed by observation under microscope.

### 2.9 Transwell invasion assay

Before Transwell assay, the cells would be transferred into serum‐free medium for 24 hours. Then, the cells were diluted with medium containing 1% FBS to a concentration of 1 × 10^5^/mL. The suspension was transferred to the upper chamber, and medium containing 10% FBS was added to the lower chamber of the Transwell following 24‐hour culture at 37°C. Then, the cells were fixed with 4% formaldehyde and stained with crystal violet. The cells were observed under a microscope and counted.

### 2.10 Western blot

A 10% sodium dodecyl sulphate polyacrylamide (SDS) gel (Willget Biotech, Shanghai, China) was used for proteins separation. Then, proteins were transferred onto a poly vinylidene difluoride (PVDF) membrane (Polyvinylidene Fluoride) following 1‐hour blocking with 5% defatted milk. Then, primary antibodies were added and incubated at 4°C over night. After washing with tris‐buffered saline tween (TBST), secondary antibody was added for 1‐hour incubation at RT. Primary antibodies, including anti‐TIMP3, anti‐E‐cadherin, anti‐N‐cadherin, anti‐Vimentin, anti‐Snail, anti‐Slug, anti‐Twist, anti‐ZEB1 and anti‐glyceraldehyde‐3‐phosphate dehydrogenase (GAPDH) were purchased from Abcam (Cambridge, UK).

### 2.11 Tumour xenografts

The Balb/C Nude mice (4‐6 weeks, male, 15‐20 g) were obtained from Charles River Laboratories, Peking, China. 5 × 10^6^ cells were resuspended in 0.1 mL of DMEM medium and subcutaneously implanted into the right flank of nude mice. Tumour volume and mice weight were recorded then.

### 2.12 Statistical analysis

All data in this research were represented as mean ± SD. Variation was compared by Student's *t* test and one‐way ANOVA. Data were considered significantly different as *P*‐value < 0.05. All data were calculated based on at least three independent experiments.

## 3 RESULTS

### 3.1 Identification of circular RNAs in human renal cell carcinoma samples

**FIGURE 1:**
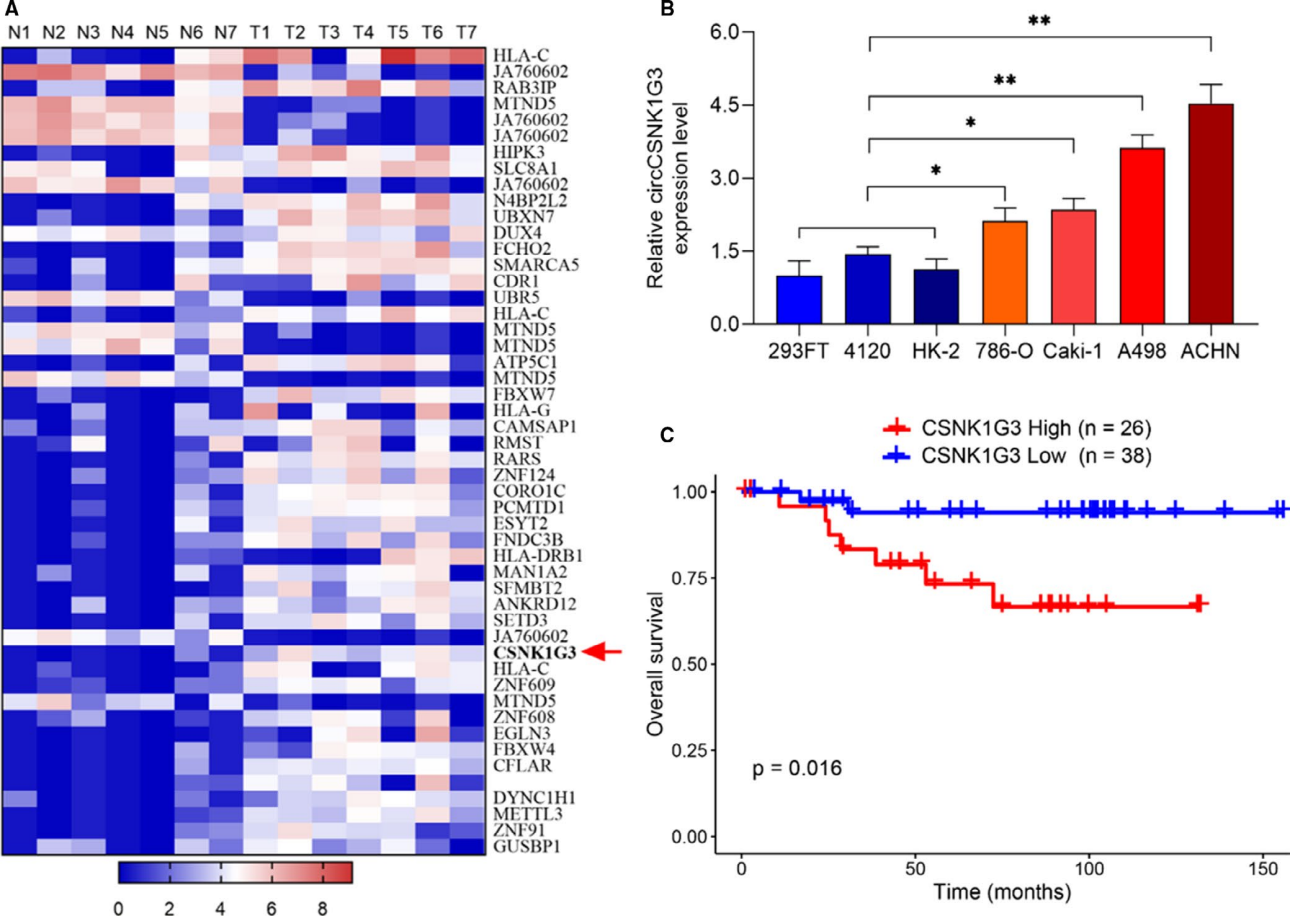
CircCSNK1G3 is highly expressed in renal cancer. A, To identify profiling of circRNAs in renal cancer, the GEO DataSets (GSE108735) were analysed. Hierarchical clustering analysis of the top 50 circRNAs that were differentially expressed between renal cell carcinoma (RCC) and normal tissues (NT) samples. We identified circCSNK1G3 was highly expressed in renal cancer. Red arrowhead denotes circCSNK1G3. B, qRT‐PCR analysis of circCSNK1G3 expression was increased in renal cancer cell lines (786‐O, Caki‐1, A498, ACHN) compared with normal renal epithelial cells (293FT, 4120, HK‐2). C, Survival analysis of 64 RCC patients having different CSNK1G3 transcripts level, including 26 patients with high expression of CSNK1G3 and 38 patients with low expression of CSNK1G3. Data from TCGA‐KICH database. *P < 0.05. **P < 0.01 [Colour figure can be viewed at wileyonlinelibrary.com]

To identify profiling of circRNAs in renal cell carcinoma (RCC), we characterized circular RNA transcripts using published RNA‐seq data from GEO DataSets (GSE108735). A landscape indicating the top 50 circRNAs that were differentially expressed between renal cell carcinoma (RCC) and normal tissues (NT) samples is shown (Figure 1A). This research focuses on the role and function of one of those circular RNA circCSNK1G3 in RCC progression. Aberrantly expressed circCNK1G3 was previous reported to contribute to the tumour growth in prostate cancer.^18^ In this research, the circCSNK1G3 showed a high expression in RCC cell lines compared with normal human renal cells lines including 293FT, 4120 and HK‐2, which implied us the regulatory potential of circCSNK1G3 in RCC (Figure 1B). In addition, the survival analysis of 64 RCC patients in TCGA‐KICH database suggested a worse prognosis in patients with high expression of CSNK1G3, including both linear mRNA and circular RNA expression, compared with patients with low expression of CSNK1G3 (Figure 1C). These data suggest that circCSNK1G3 may play an important role in RCC.

### 3.2 The characteristics of the circular RNA circCSNK1G3

**FIGURE 2:**
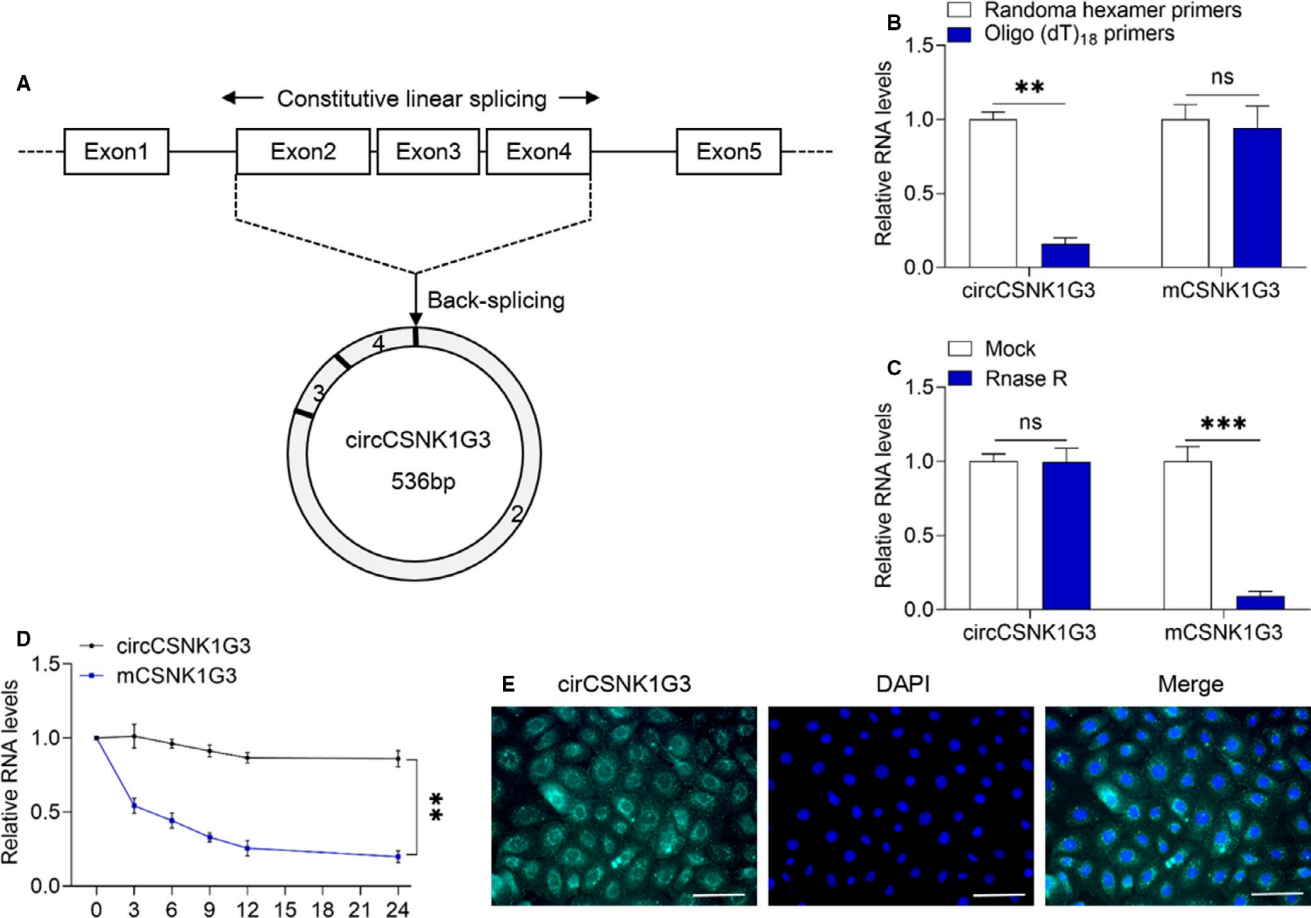
The characteristics of the circular RNA circCSNK1G3. A, Scheme illustrating the production of circCSNK1G3. B, The relative RNA reverse transcripted with random hexamer or oligo (dT)18 primers were measured using qRT‐PCR. C, The circCSNK1G3 and mCSNK1G3 expression measured by qRT‐PCR after Rnase R treatment. D, The expression of circCSNK1G3 and mCSNK1G3 measured by qRT‐PCR after actinomycin D treatment at different time in ACHN cells. E, circCSNK1G3 were detected by RNA FISH. Scale bar, 50 μm. *P < 0.05. **P < 0.01 [Colour figure can be viewed at wileyonlinelibrary.com]

CircCSNK1G3 derived from exons 2, 3 and 4 of the CSNK1G3 gene [circbase ID: hsa_circ_0001522, termed circCSNK1G3] was chosen for subsequent research (Figure 2A). In order to confirm the characteristics of circCSNK1G3, we carried out qRT‐PCR on RNA from RCC derived ACHN cells using random hexamer or oligo (dT)18 primers. Oligo (dT) 18 primers amplified mCSNK1G3 effectively but not circCSNK1G3 (Figure 2B), showing that circCSNK1G3 did not have poly‐A tail. Moreover, circCSNK1G3 was resistant to RNase R who can digest linear RNA, indicating that circCSNK1G3 is not linear but circular (Figure 2C). After transcription inhibition, circCSNK1G3 showed a longer half‐life and was more stable than mCSNK1G3 (Figure 2D). Furthermore, fluorescence in situ hybridization (FISH) results indicated that circCSNK1G3 is predominantly distributed in cytoplasm (Figure 2E). These results demonstrated that circCSNK1G3 which was highly expressed in RCC is a circular and stable transcript of CSNK1G3 with a predominant cytoplasmic distribution.

### 3.3 CircCSNK1G3 inhibits renal cell carcinoma progression in vitro

**FIGURE 3:**
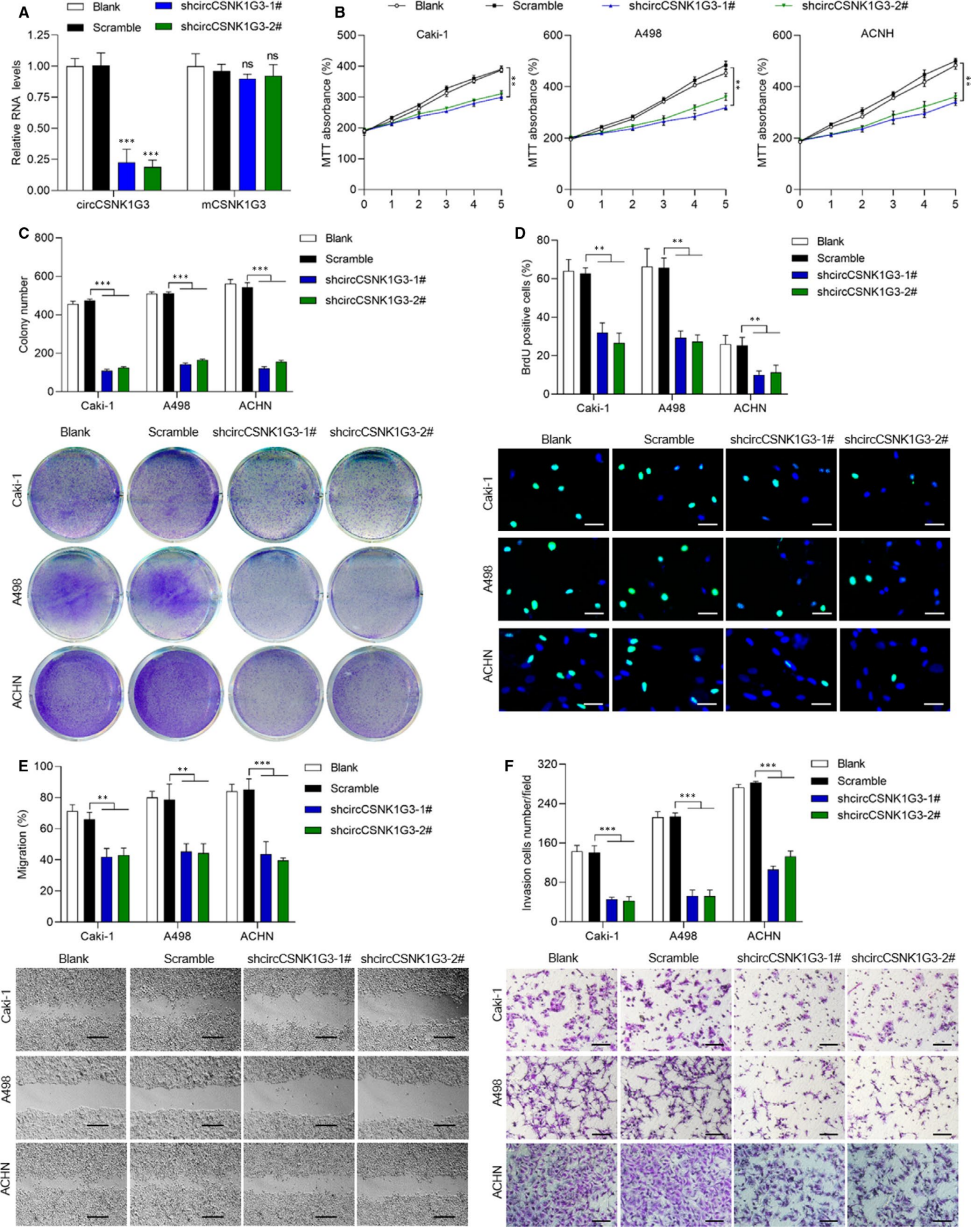
Knockdown of circCSNK1G3 obviously inhibits RCC cells proliferation, migration and invasion. A, Left, the knockdown efficiency of shRNA against circCSNK1G3 was determined by qRT‐PCR in ACHN. Right, the effect of shRNA against circCSNK1G3 on CSNK1G3 mRNA expression. B, MTT assays revealed that down‐regulation of circCSNK1G3 significantly reduced the growth rate in RCC cell lines including Caki‐1, A498 and ACHN. C, Colony formation assay showed that down‐regulation of circCSNK1G3 reduced the mean colony number. Bottom, representative images of Caki‐1, A498 and ACHN. D, BrdU immunofluorescence staining showed that down‐regulation of circCSNK1G3 reduced cells proliferate capacities. Bottom, representative images of Caki‐1, A498 and ACHN. Scale bars, 100μm. E, Wound healing assay showed that down‐regulation of circCSNK1G3 reduced cell migration rate. Bottom, representative images of Caki‐1, A498 and ACHN migration measured at 48 h. Scale bars, 50 μm. F, Transwell invasion assay showed that down‐regulation of circCSNK1G3 reduced cell invasion number. Bottom, representative images of Caki‐1, A498 and ACHN invasion across the transwell measured at 48 h. Scale bars, 20 μm. Data are shown as the mean ± standard deviation (n = 3) and representative of three independent experiments. *P < 0.05. **P < 0.01 (Student's *t* test) [Colour figure can be viewed at wileyonlinelibrary.com]

In order to explore the role of circCSNK1G3 in RCC, circCSNK1G3 was specifically knocked down without affecting the expression of CSNK1G3 mRNA (Figure 3A). After circCSNK1G3 knockdown, the proliferation of RCC cells significantly decreased (Figure 3B) which was confirmed by subsequent colony formation assay (Figure 3C) and BrdU immunofluorescence staining (Figure 3D). In addition, both cell migration and cell invasion capacities were inhibited after circCSNK1G3 knockdown (Figure 3E,F). These results showed the carcinogenesis of circCSNK1G3 to promote cell proliferation, migration and invasiveness of RCC cells, which contributes to the tumour growth and metastasis in RCC.

### 3.4 CircCSNK1G3 promotes tumour growth and metastasis in RCC via miR‐181b‐TIMP3

**FIGURE 4:**
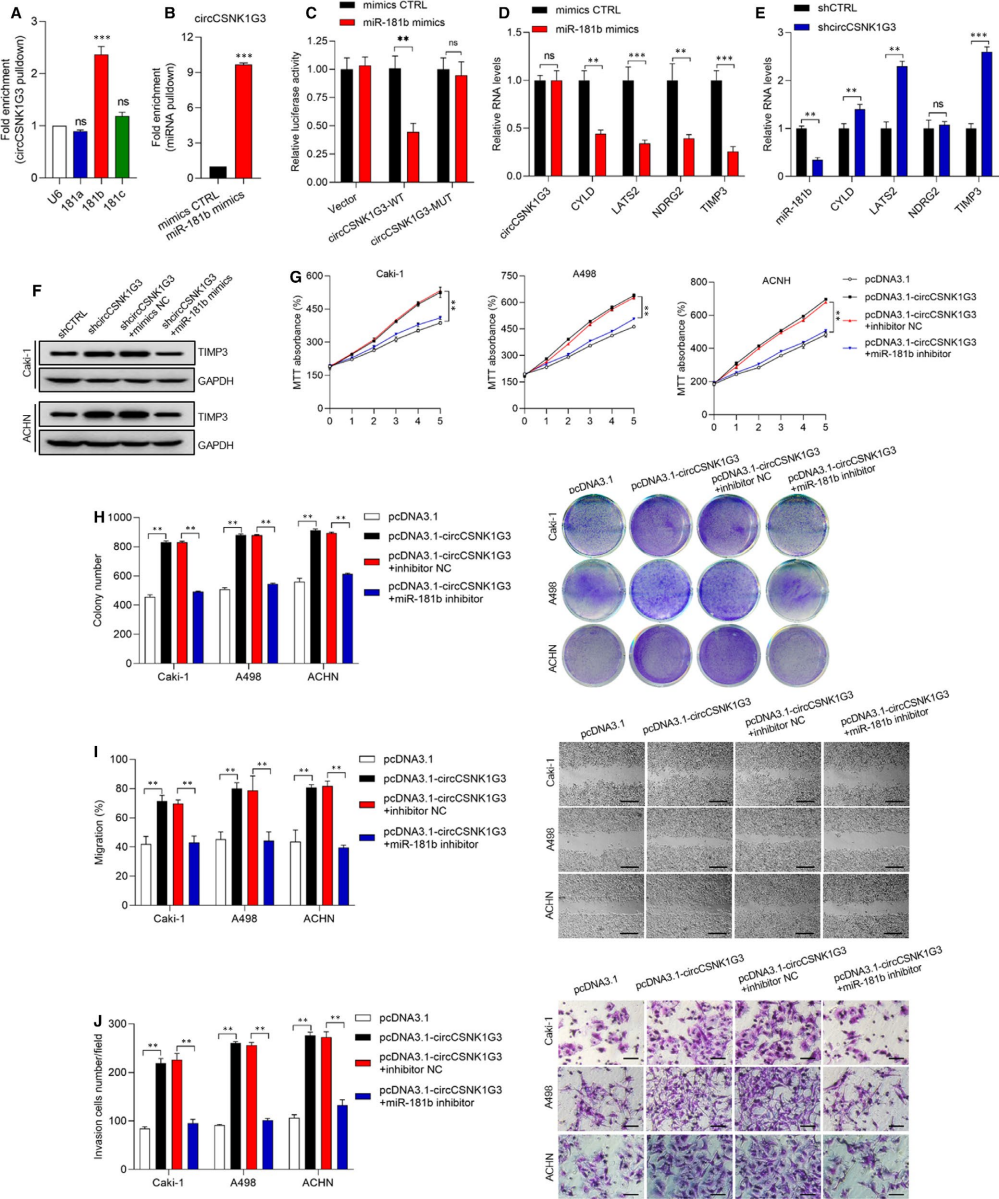
CircCSNK1G3 interacts with miR‐181b to promote RCC cells proliferation, migration and invasion. A, RNA pull‐down using biotinylated circCSNK1G3, miRNAs were detected by qRT‐PCR. U6 was used as controls. B, RNA pull‐down using biotinylated miR‐181b mimics. circCSNK1G3 was detected by qRT‐PCR. Abundance levels were normalized to biotinylated mimic controls. C, Luciferase report assay representing the interaction between circCSNK1G3 and miR‐181b. D, Overexpression miR‐181b mimics and measuring the expression of its respective targets. As expected, TIMP3 was down‐regulated upon overexpression of miR‐181b. E, TIPM3 was up‐regulated by circCSNK1G3/miR‐181b. F, Western blot showed that overexpression of miR‐181b mimics could rescue the expression of TIMP3 upon cicrCSNK1G3 loss. G and H, Overexpression of circCSNK1G3 promotes cells proliferation and rescued by miR‐181b inhibitor via MTT assay and colony formation assay. I, Overexpression of circCSNK1G3 promotes cells migration and rescued by miR‐181b inhibitor via wound healing assay. Bottom, representative images of ACHN migration measured via wound healing assay at 48 h. Scale bars, 50 μm. J, Overexpression of circCSNK1G3 promotes cells invasion and rescued by miR‐181b inhibitor via transwell invasion assay. Bottom, representative images of ACHN invasion across the transwell measured at 48 h. Scale bars, 20 μm. Data are shown as the mean ± standard deviation (n = 3) and are representative of three independent experiments. *P < 0.05. **P < 0.01 (Student's *t* test) [Colour figure can be viewed at wileyonlinelibrary.com]

Previous research has indicated that the circCSNK1G3 could promote cell proliferation by interacting with miR‐181 in prostate cancer.^18^ In order to confirm the effects of circCSNK1G3 on miR‐181b in RCC, we performed the RNA pull‐down assay using biotinylated circCSNK1G3. The results showed an obvious interaction between circCSNK1G3 and miR‐181b (Figure 4A) which was confirmed in the subsequent RNA pull‐down assay using biotinylated miR‐181b mimics (Figure 4B). Subsequently, the luciferase report assay was carried out to confirm the direct interaction between circCSNK1G3 and miR‐181b (Figure 4C).

To further investigate the role of miR‐181b in RCC, we detected the expression of several tumour suppressor gene, including CYLD, LATS2, NDRG2 and TIMP3, after miR‐181b overexpression. The RT‐PCR results showed that the overexpression of miR‐181b significantly impaired those tumour suppressor gene expressions (Figure 4D). To the contrary, the decrease of miR‐181b induced by circCSNK1G3 silencing notably promoted the expression of CYLD, LATS2 and TIMP3 except NDRG2 (Figure 4E). The results indicated that TIMP3, a common target of miR‐181b‐5p, was the most strikingly increased gene in these genes after the inhibition of circCSNK1G3 and miR‐181b (Figure 4E). Then, the Western blot results also showed the increased TIMP3 protein level after circCSNK1G3 silencing, which can be reversed by miR‐181b overexpression (Figure 4F). In fact, TIMP3 was a widely studied tumour suppressor in many cancer types. Previous study has demonstrated that miR‐181b promoted carcinogenesis by targeting TIMP3 in hepatocellular carcinoma.^31^ And our subsequent study also provided further exploration of the effects of TIMP3 in RCC.

In addition, we evaluated the carcinogenesis of RCC cells under different circCSNK1G3 expression level. After circCSNK1G3 overexpression, the cell proliferation (Figure 4G), colonization (Figure 4H), migration (Figure 4I) and invasiveness (Figure 4J) were all apparently increased, and this carcinogenesis induced by circCSNK1G3 got lost after miR‐181b inhibition. These data suggested that circCSNK1G3 acted as an oncogenic role to promote the tumour growth and metastasis in RCC by positively regulating miR‐181b and thus subsequently impaired the expression of other tumour suppressor gene.

### 3.5 CircCSNK1G3 promotes the EMT process by inhibiting TIMP3 in renal cell carcinoma

**FIGURE 5:**
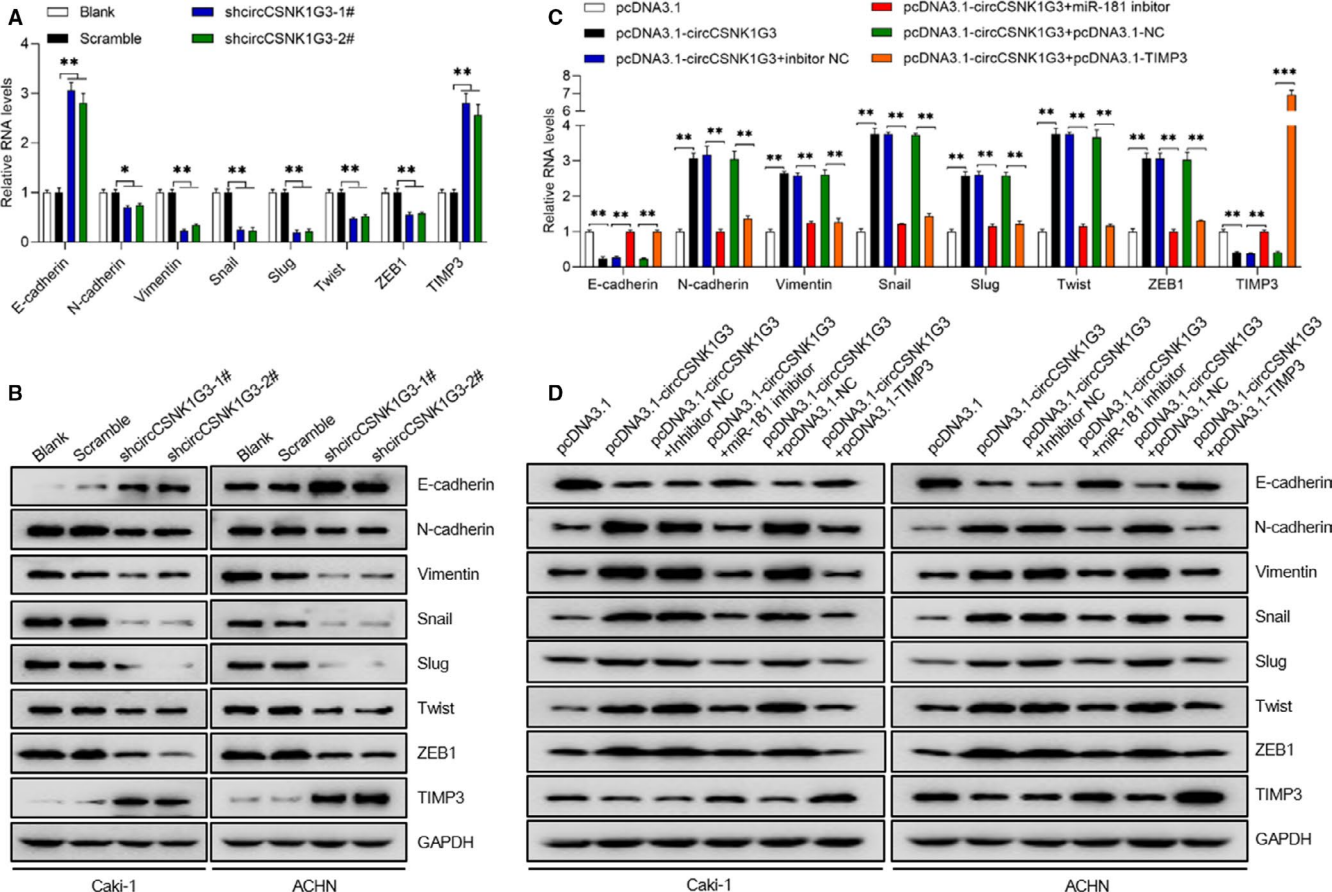
CircCSNK1G3 facilitates epithelial to mesenchymal transition in RCC cells via miR‐181b‐TIMP3 pathway. A and B, Down‐regulation of circCSNK1G3 reduced the mRNA and protein expression level of EMT‐associated markers measured by qRT‐PCR or Western blot in Caki‐1 and ACHN after transfection with Scramble or shcircCSNK1G3‐1#, shcircCSNK1G3‐2#. C and D, mRNA and protein level of EMT‐associated markers measured by qRT‐PCR and Western blot. Data are shown as the mean ± standard deviation (n = 3) and are representative of three independent experiments. *P < 0.05. **P < 0.01 (Student's *t* test) [Colour figure can be viewed at wileyonlinelibrary.com]

As circCSNK1G3 promoted cell migration and invasion in RCC cells, it can be assumed that circCSNK1G3 to some extent modified the cell phenotype into a more aggressive form. Epithelial to mesenchymal transition (EMT) is widely known to be closely associated with cancer cell metastasis; therefore we examined a variety of EMT‐associated marker in RCC cells. The results showed that the circCSNK1G3 knockdown notably increased the expression of epithelial cell adhesion factor E‐cadherin both in Caki‐1 cells and ACHN cells (Figure 5A,B). To the contrary, N‐cadherin and Vimentin were decreased after circCSNK1G3 silencing (Figure 5A,B). Besides, the EMT‐promoting factors, including Snail, Slug, Twist and ZEB1, got significantly decreased after circCSNK1G3 silencing (Figure 5A,B).

Subsequently, the circCSNK1G3 was overexpressed in Caki‐1 cells and ACHN cells and both the two cell lines represented a enhanced EMT process induced by circCSNK1G3. Moreover, the inhibition of miR‐181b notably reversed the EMT‐promoting effects caused by overexpressed circCSNK1G3. More important, overexpressed TIMP3 can also reverse the EMT‐promoting effects caused by overexpressed circCSNK1G3 (Figure 5C,D). Combined with the previous results, a suppressive effect of circCSNK1G3 on TIMP3 through up‐regulating miR‐181b expression as well as promoting the EMT process can be concluded.

### 3.6 Decreased circCSNK1G3 restrains tumour growth in renal cell carcinoma

**FIGURE 6:**
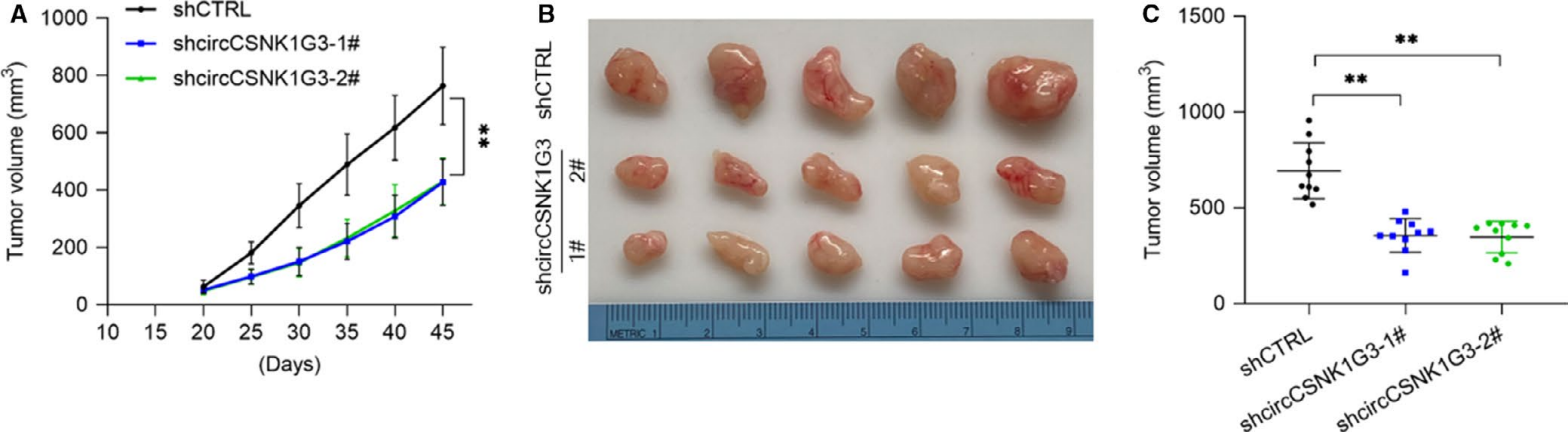
Down‐regulation of circCSNK1G3 inhibits the growth of renal cancer cells in vivo. A, NOD/SCID mice (8 wk old) were inoculated subcutaneously with ACHN cells (1 × 10^7^ per tumour) pre‐transfected with shCTRL or two shcircCSNK1G3. Tumour volumes were measured at different time points. Data points are presented as mean volume ± SD values. B and C, Comparative statistics of tumour end volumes and images of excised tumours from five NOD/SCID mice at 45 d. **P < 0.01 (Student's *t* test) [Colour figure can be viewed at wileyonlinelibrary.com]

To better understand the role of circCSNK1G3 in RCC progression, ACHN cells, wild‐type or circCSNK1G3 knockdown, were subcutaneously injected into NOD/SCID mice for tumour xenografts. It was shown that the knockdown of circCSNK1G3 significantly impaired tumour growth in vivo (Figure 6A). The final tumour volume was significantly dropped when circCSNK1G3 was knocked down (Figure 6B,C), indicating a significant promotive effect of circCSNK1G3 on the development of RCC in vivo. Taken together, we characterized the circular RNA circCSNK1G3 in RCC progression. The results showed circCSNK1G3, as an oncogenic role, suppressed TIMP3 by up‐regulating miR‐181b, thereby promoted the EMT process, and finally contribute to the tumour growth and metastasis in RCC.

## 4 DISCUSSION

In this research, we screened a variety of circular RNAs which are differentially expressed between renal cell carcinoma and normal tissue sample through published RNA‐seq data. We tried to explore the role and mechanism of the up‐regulated circCSNK1G3 in RCC progression. We also declared that the increased circCSNK1G3 promoted tumour growth and metastasis by positively regulating miR‐181b and facilitating EMT process in RCC.

Casein kinase I (CKI) γ3 gene (CSNK1G3), located on chromosome 5q23, is a member of CK1 family, which is a ubiquitous Ser/Thr protein kinase found in the nuclei, cytoplasm and membrane fraction of eukaryotic cells.^32^ CSNK1G3 was important for protein phosphorylation. It has been reported that the abnormally expression of CSNK1G3 play a critical role in human disease.^33^, ^34^ In particular, it has been reported that the silence of CSNK1G3 caused significant cell killing in human renal carcinoma cells and thus decreased the phosphorylation of Akt and ribosomal protein S6.^35^

Interestingly, in this research the expression of circCSNK1G3 was significantly increased in renal cell carcinoma than in normal renal cells. The expression and function of circCSNK1G3 in RCC have never been studied. Then, we investigated the role and mechanism of increased circCSNK1G3 in human RCC progression. We found that the up‐regulation of circCSNK1G3 contributed to the cell proliferation, migration and invasiveness in RCC. Moreover, the carcinogenesis of circCSNK1G3, at least partly, was achieved by up‐regulating miR‐181b. Both inhibition of miR‐181b and knockdown of circCSNK1G3 impaired tumour growth in RCC. Importantly, circular RNA was widely reported to sponging miRNAs to regulate tumour growth in a variety of cancers. But our data indicated that circCSNK1G3 can positively regulate miR‐181b by directly interacting with miR‐181b in RCC, which expanded the comprehension of the role and mechanism of cricRNAs in tumour progression.

MiR‐181b is a member of miR‐181 family containing miR‐181a/b/c/d that was initially described as a promoter of haematopoietic differentiation^36^ and was shown to serve as dual‐role regulator in the development of human cancers.^37^ Previous researches demonstrated that miR‐181b have both cancer‐promoting and anti‐cancer effects in different cancer types.^38^, ^39^ Recently, miR‐181b has been found to contribute to tumour growth and metastasis by suppressing the anti‐tumour gene TIMP3 and induced EMT process in multiple cancers, including hepatocellular carcinoma and breast cancer.^29^, ^31^, ^40^ The zinc finger proteins, Snail, Slug, ZEB1 and the basic helix‐loop‐helix factor Twist were EMT regulatory proteins^41^ and were reported to be regulated by miR‐181b in human bronchial epithelial cells.^42^ In our study, we discovered a strong positive regulation of circCSNK1G3 on miR‐181b in RCC cells. The tumour‐promoting effects caused by circCSNK1G3 got inhibited after miR‐181b inhibition, revealing the anti‐cancer effect of circCSNK1G3 obtained by up‐regulating miR‐181b as well as the oncogenic role of miR‐181b in RCC. Moreover, the overexpression of miR‐181b significantly repressed the expression of anti‐tumour genes, such as TIMP3 which regulates the EMT process. Notably, most previous researches indicated that circular RNAs act as miRNAs sponge to inhibit miRNAs expression.^43^, ^44^ But our data demonstrated that the circCSNK1G3 up‐regulated miR‐181b by directly interacting with miR‐181b in RCC. Recently, several researches used computational analysis to show that circRNAs are not only the sponges of miRNAs but also have other effects, which may provide us new insight into the mechanism of circular RNA in biological regulation.^45^, ^46^

In addition, the decrease of circCSNK1G3 also facilitated the expression of TIMP3, a member of tissue inhibitors of metalloproteinases (TIMP) family, which regulate a vast range of cell surface proteins and resulting in prominently effects on tumour growth and cancer metastasis.^47^, ^48^ TIMP3 acts as a tumour suppressor, and the loss of TIMP3 has been found in several human cancers, including renal cell carcinoma, and has been proved to promote tumour metastasis.^49^, ^50^ Here, our data showed the loss of TIMP3 caused by circCSNK1G3/miR‐181b in renal cell carcinoma promoted the cell proliferation, colonization, migration and invasion. The silencing of circCSNK1G3 notably increased the level of TIMP3 protein and inhibited the carcinogenesis. However, the promotive effects of circCSNK1G3 silencing on TIMP3 were lost after miR‐181b overexpression. The overexpression of miR‐181b significantly repressed the expression of TIMP3 induced by the knockdown of circCSNK1G3. These data indicated that circCSNK1G3/miR‐181b axis impaired the expression of TIMP3, which also suggested the oncogenic role of circCSNK1G3/miR‐181b axis in RCC.

More important, our data also discovered that TIMP3 repressed the EMT process in RCC. Epithelial‐mesenchymal transition (EMT) was an important developmental programme which has been shown to play a crucial role in cancer development and metastasis.^51^, ^52^ Tumour metastasis is the main cause of poor prognosis in RCC, and a large proportion of cancer patients with RCC have cancer metastasis, which terms to be a big challenge of RCC therapy.^53^ Tumour cells lose the adhesion properties and thus invade to other tissues through EMT process.^54^ During EMT process, E‐cadherin is an important epithelial cell adhesion factor whose reduction leads to loss of intercellular adhesion.^55^ On the other side, increased N‐cadherin, Vimentin, Snail, Slug, Twist and ZEB1 are declared to contribute to EMT process.^42^ Herein, we found that the loss of TIMP3 induced by the knockdown of circCSNK1G3 notably repressed the EMT process in RCC cells, indicating that circCSNK1G3 can lead to tumour metastasis by promoting the EMT process in RCC.

Taken together, our research revealed that circCSNK1G3 suppressed the tumour growth and metastasis, at least partly, by up‐regulating miR‐181b in human renal cell carcinoma and hold the promise of being developed as a diagnostic and prognostic biomarker in RCC patients. Moreover, the regulatory role of circCSNK1G3/miR‐181b/TIMP3 axis was preliminarily demonstrated in RCC. These findings indicate an important progress in our understanding of renal cell carcinoma progression and lay the foundation for further functional, diagnostic and therapeutic study of circular RNAs in RCC.

## CONFLICTS OF INTEREST

The authors declare no conflict of interest.

## AUTHOR CONTRIBUTIONS

**Wen Li:** Conceptualization (equal); Investigation (equal); Methodology (equal); Project administration (equal). **Yang‐Yi‐Yan Song:** Formal analysis (equal); Visualization (equal). **Ting Rao:** Investigation (equal); Methodology (equal). **Weimin Yu:** Formal analysis (equal); Visualization (equal). **Yuan Ruan:** Formal analysis (equal); Visualization (equal). **Jin‐Zhuo Ning:** Data curation (equal); Methodology (equal). **Xiao‐Bing Yao:** Investigation (equal); Methodology (equal). **Song‐Yi‐Sha Yang:** Data curation (equal); Writing‐review & editing (equal). **Fan Cheng:** Conceptualization (equal); Funding acquisition (equal); Resources (equal); Writing‐original draft (equal); Writing‐review & editing (equal).

## ETHICS APPROVAL

Approved by the Renmin Hospital of Wuhan University.

## Data Availability

The data used to support the findings of this study are available from the corresponding author upon request.
